# Obstructive uropathy secondary to bilateral ureteroinguinoscrotal herniation

**DOI:** 10.1590/S1677-5538.IBJU.2015.0006

**Published:** 2016

**Authors:** Oladapo Feyisetan, Michael S Floyd, Azi Samsudin

**Affiliations:** 1 St Helens & Knowsley Teaching Hospitals NHS Trust – Urology Prescot, United Kingdom of Great Britain and Northern Ireland

## CASE PRESENTATION

**Figure 1:**
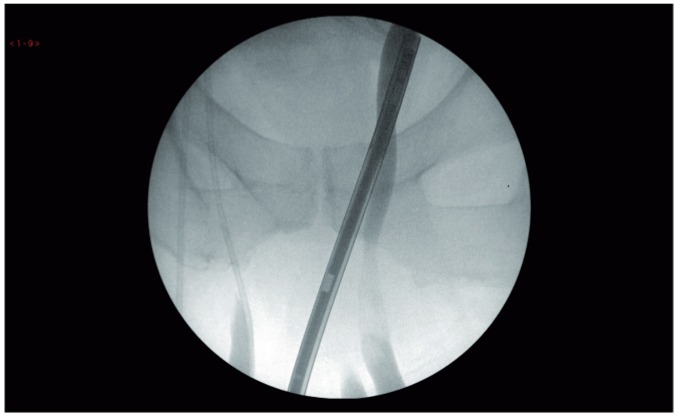
Retrograde pyelograph showing the lower ends of both ureters looping below the pubic arch before ascending towards the kidneys.

**Figure 2:**
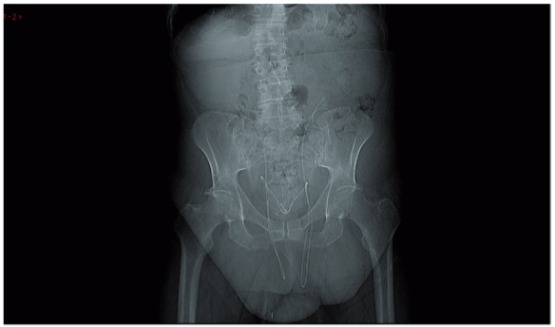
CT-KUB scout film. The stents outline the unusual anatomical passage of both ureters. The upper coil of the stents shows the renal pelvises have been pulled down to L4/L5vertebral level.

**Figure 3:**
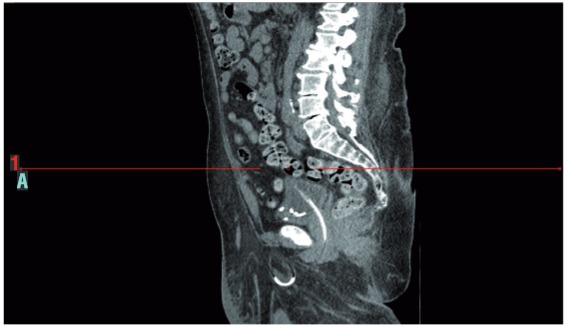
In this sagittal section of the CT-KUB, the left ureter, as outlined by the stent, can be seen passing through the neck of the hernia sac.

A 55 year old man presented with acute renal failure. He was grossly overweight with a BMI of 48 and had a past history of sleep apnoea, chronic lymphoedema and left ventricular dysfunction. Physical examination revealed a pendulous abdomen which extended to his knees and bilateral, irreducible inguinoscrotal hernias. Blood samples on admission revealed a serum creatinine of 187umol/l and an eGFR of 33ml/min. CT urogram demonstrated bilateral hydronephroureter to the level of the vesico-ureteric junction. The ureters were found to be tortuous and appeared to extend below the bladder before looping back up into the bladder. The absence of contrast within the ureters made the position of the lower ureter difficult to determine. Subsequent MAG-3 renogram showed a split function of 39/61% with a right sided preponderance. Both kidneys were slow to peak and showed negligible drainage. Intraoperative retrograde pyelograghy showed the ureters to be grossly elongated, looping down bilaterally through the hernial sacs within the scrotum before returning up to the kidneys (Figure-1). Conventional double pig tail ureteric stents were found to be not long enough to span the distance between the bladder and renal pelvis and 75cm-long ileal conduit stents were used successfully (Figure 2 and 3).

## DISCUSSION

Sliding inguinal hernias which contain the ureter have been reported in the literature (1, 2). Case reports also exist of associated ureteric obstruction and with resultant ureterohydronephrosis (3-5). Definitive management involves repair of the hernia with care taken to preserve the ureters. These patients are usually morbidly obese (6) and are likely to have co-morbidities that make surgery a high risk (3-5).
